# The Relationship between the Expression of Fatty Acyl Desaturase 2 (*fads2*) Gene in Peripheral Blood Cells (PBCs) and Liver in Gilthead Seabream, *Sparus aurata* Broodstock Fed a Low n-3 LC-PUFA Diet

**DOI:** 10.3390/life10070117

**Published:** 2020-07-19

**Authors:** Shajahan Ferosekhan, Serhat Turkmen, Hanlin Xu, Juan Manuel Afonso, Maria Jesus Zamorano, Sadasivam Kaushik, Marisol Izquierdo

**Affiliations:** 1 Aquaculture Research Group (GIA), IU-ECOAQUA, Universidad de Las Palmas de Gran Canaria, 35214 Telde, Spain; turkmen@uab.edu (S.T.); hanlinxuulpgc@outlook.com (H.X.); juanmanuel.afonso@ulpgc.es (J.M.A.); mariajesus.zamorano@ulpgc.es (M.J.Z.); sachi.kaushik@ulpgc.es (S.K.); marisol.izquierdo@ulpgc.es (M.I.); 2 ICAR-Central Institute of Freshwater Aquaculture, Bhubaneswar 751002, India; 3 Department of Biology, University of Alabama at Birmingham, Birmingham, AL 35294, USA

**Keywords:** biomarker, broodstock, fatty acyl desaturase (*fads2*), fatty acid, lipid metabolism

## Abstract

The principle aim of this study is to elucidate the relationship between the fatty acid desaturase 2 gene (*fads2*) expression pattern in peripheral blood cells (PBCs) and liver of gilthead seabream (GSB), *Sparus aurata* broodstock in order to determine the possible use of *fads2* expression as a potential biomarker for the selection of broodstock. This selection could be utilized for breeding programs aiming to improve reproduction, health, and nutritional status. Passive Integrated Transponder (PIT)-tagged GSB broodstock (Male-1.22 ± 0.20 kg; 44.8 ± 2 cm and female-2.36 ± 0.64 kg; 55.1 cm) were fed a diet containing low levels of fish meal and fish oil (EPA 2.5; DHA 1.7 and n-3 LC-PUFA 4.6% TFA) for one month. After the feeding period, *fads2* expression in PBCs and liver of both male and female broodstock were highly significantly correlated (r = 0.89; *p* < 0.001). Additionally, in male broodstock, liver *fads2* expression was significantly correlated (*p* < 0.05) to liver contents in 16:0 (r = 0.95; *p* = 0.04) and total saturates (r = 0.97; *p* = 0.03) as well as to 20:3n–6/20:2n–6 (r = 0.98; *p* = 0.02) a Fads2 product/precursor ratio. Overall, we found a positive and significant correlation between *fads2* expression levels in the PBCs and liver of GSB broodstock. PBCs *fads2* expression levels indicate a strong potential for utilization as a non-invasive method to select animals having increased fatty acid bioconversion capability, better able to deal with diets free of fish meal and fish oil.

## 1. Introduction

**Figure 1:**
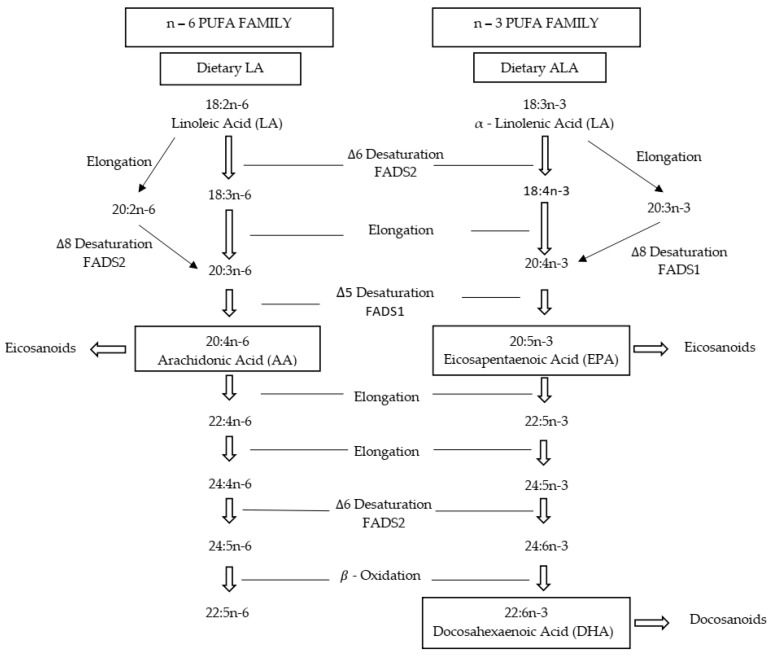
Endogenous biosynthesis pathway of n-6 and n-3 long chain polyunsaturated fatty acids (LC-PUFA) from linoleic acid (LA) and α-linolenic acid (ALA) substrate through desaturation (Fads), elongation (Elvol), and β-oxidation in fish.

The ability of teleosts to biosynthesize long-chain (≥C20) polyunsaturated fatty acids (LC-PUFA), such as arachidonic acid (ARA, 20:4n-6), eicosapentaenoic acid (EPA, 20:5n-3) or docosahexaenoic acid (DHA, 22:6n-3), from 18 carbon PUFA substrate, linoleic acid (LA; 18:2n-6), and α-linolenic acid (ALA; 18:3n-3) is limited [1,2,3]. Knowledge on this is of vital importance in order to quantify the dietary requirements for essential fatty acids (EFA) in farmed fish and to tailor the fatty acid composition of the fish fillet for human consumption [4,5]. The fatty acid biosynthesis comprises several steps catalyzed by fatty acyl desaturases (Fads1-∆5 and Fads2-∆6 desaturase) and an elongation of very long chain fatty acids proteins (Elovl), where ∆6 desaturase is a major rate-limiting step in the fatty acid biosynthesis pathway (Figure 1) [3,6]. The ∆6 desaturase introduces new double bonds into fatty acyl chains, essential to the first step in the LC-PUFA biosynthesis and is encoded by the fatty acyl desaturase gene (*fads*). Carnivorous species such as felids have very limited ability to synthesize LC-PUFA [7], as well as teleost fish from higher trophic levels, which consequently have a dietary requirement for pre-formed LC-PUFA [8]. In general, the majority of vertebrates lack the capacity to biosynthesize PUFA de novo, and different teleost species have a wide range of bioconversion capability for converting dietary C18 PUFA to LC-PUFA [1,8,9]. Among teleosts, freshwater fish and especially fish from lower trophic levels are considered capable of bioconversion of dietary C18 PUFA such as linoleic acid (LA; 18:2n-6) and α-linolenic acid (ALA; 18:3n-3) into LC-PUFA, whereas marine teleosts have limited ability to biosynthesize the LC-PUFA from LA and ALA substrates [1,8,10]. This is imputed to the lower activity of *Fads*, which is due to the lower expression of the *fads* gene in marine fish compared to freshwater fish [6,11,12,13]. Low expression or absence of the *fads* gene is the main reason for the reduced synthesis of LC-PUFA in marine fish [6,8,14]. Fads has been characterized and studied in several organisms from lower invertebrates to higher vertebrates [14]. Mammals possess two FADS enzymes termed FADS1 and FADS2, which are involved in different steps of desaturation activity. FADS1 is a ∆5 desaturase, whereas FADS2 is a ∆6 desaturase with the ability to utilize C18 substrates [15] in order to biosynthesize ARA, EPA, and DHA. In marine fish, it is well documented that the biosynthesise of n-3 LC-PUFA is initiated by the delta 6 fatty acid desaturase (Δ6 Fads), which acts on ALA or LA to produce EPA and DHA or ARA through several steps of desaturation, elongation, and β-oxidation (Figure 1).

Fads2 from most of the teleost species show ∆6 desaturase activity; its activity was also identified in many species such as zebra fish, *Danio rerio* [16], gilthead seabream (GSB) (*Sparus aurata*) [1,17,18,19,20], European seabass (*Dicentrarchus labrax*) [21,22], black seabream (*Acanthopagrus schlegeli*) [23], meagre (*Argyrosomus regius*) [24], orange-spotted grouper (*Epinephelus coioides*) [25], nibe croaker (*Nibea mitsukurii*) [26], rabbit fish (*Siganus canaliculatus*) [27], rainbow trout (*Oncorhynchus mykiss*) [19], Atlantic salmon (*Salmo salar*) [2,28], and Atlantic cod (*Gadus morhua*) [29]. Analysis of the different studies on enzyme activities of several species shows that many species have several desaturase activities. For example, zebrafish Δ6 desaturase, which was the first functionally characterized Fads2 in a teleost, showed three desaturase activities: Δ5, Δ6, and Δ8 [16]. Similarly, dual Δ6 and Δ8 desaturase activity has been reported in other teleost Fads2 including GSB [1,19], rabbitfish [1,8,27], rainbow trout [19], turbot [19], Atlantic salmon [1,2], and grouper [25]. The *fads2* gene expression in different tissues has been studied in several marine fish species although the importance and implication of the *fads2* gene in fish growth and reproduction has not been deeply studied besides a few studies by our own research group [30,31,32,33].

Lipid metabolism and fatty acid synthesis occurs mainly in the liver, then fatty acids are transported through the blood to the rest of the tissues in the body for utilization in diverse physiological functions [34]. Knowing the activity and expression of Δ6 desaturase and *fads2* in liver is paramount in order to ascertain FA’s synthesis and mobilization to various tissues. The measurement of *fads2* expression in the liver, however, requires sacrificing the animal and it is a major constraint for working on live fish. Hence, it is necessary to develop a non-invasive method to study the *fads2* expression in fish. It is well known that blood is the most accessible tissue to study the well-being of an animal to predict or assess the physiological conditions of other tissues by means of a non-invasive manner. In humans, peripheral blood cells (PBCs) are widely used to check the health status by analyzing the gene expression pattern in peripheral blood and to predict the same gene expression in non-accessible tissues like brain, liver, gonad, kidney, lungs etc. In various human studies, expression of many health status indicative genes in PBCs show significant correlations to the respective gene expression in different other organ tissues [35,36,37,38].

The presence of a nucleus and functional mitochondria in the red blood cells of fish makes blood a promising tissue to analyze the gene expression and metabolic responses in an integrative and non-invasive manner. Some studies with GSB have addressed the issue to determine whether samples collected without sacrificing animals provide a reliable measure of mitochondrial functioning and energy metabolism at the level of the whole organism [39]. In one such study using transcriptome analysis, it was observed that whole blood cell gene expression of hypoxia induced GSB juveniles and reflected the metabolic condition and mitochondrial respiration of target tissues [39]. Such an approach could be used in GSB broodstock to understand the relationship between *fads2* expression in PBCs and the liver to identify the potential of using *fads2* as a biomarker to assess the role of n-3 LC-PUFA in reproductive performance. The correlation between PBCs and hepatic *fads2* expression has not been studied so far in any fish or even in other animals. However, relative *fads2* expression between various tissues has been studied and reported in humans [40] as well as in some fish such as the Japanese seabass (*Lateolabrax japonicus*) or the golden pompano (*Trachinotus ovatus*) [41,42]. In Japanese seabass, *fads2* expression was found to be higher in the brain, eyes, liver, and intestine compared to the kidney, skin, muscle, gill, spleen, stomach, blood, and heart [41]. In golden pompano, the *fads2* expression level was higher in the brain, in comparison to the small intestine and the female gonads, whereas lower expression levels were observed in the fin, gill, blood, and kidney [42].

To our knowledge, there is no known information on the comparative relationship between blood and liver *fads2* expression patterns, nor potential differences in male and female fish. The blood *fads2* gene expression as a biomarker could be useful to study not only the fatty acid metabolism in fish but also to assess the nutritional quality of fish fillet [32,33,43] or the reproductive performance in fish [31,33]. The *fads2* expression pattern in animals are species- and sex-specific [44,45,46,47]. In an earlier study, we observed that *fads2* expression in the blood of GSB broodstock was higher in females than in males [31] and that the GSB exhibits great variations in the *fads2* expression among individuals. Therefore, the present study was undertaken to relate the expression pattern of PBCs and hepatic *fads2* in fish to elucidate the relationship in GSB broodstock to identify the potential of selecting PBCs *fads2* as a biomarker to understand the reproductive performance of fish. A second objective was to analyze the hepatic fatty acid profile of GSB broodstock fish exhibiting different *fads2* expression levels in peripheral blood cells and liver.

## 2. Materials and Methods

### 2.1. Broodstock Management and Feeding

Gilthead seabream broodstock were individually PIT tagged (EID Iberica SA-TROVAN, Madrid, Spain) and maintained in a 10 ton square tank. Male (1.22 ± 0.20 kg; 44.8 ± 2 cm; 4 fish) and female (2.36 ± 0.64 kg; 55.1 ± 2 cm; 16 fish) broodstock were maintained in the same tank. Both male and female broodstock were studied for their *fads2* expression pattern in blood cells and liver. The tank was supplied with seawater (37 g L^−1^ salinity, 17.0–20.0 °C) at a daily water exchange rate of 600% and fish were maintained under natural photoperiod. All the broodstock were fed 1% body weight twice a day for one month with a diet containing low levels of fish meal (FM, 5%) and fish oil (FO, 3%) (Table 1). The experimental diet was produced by Biomar [48] and re-pelletized at the feed manufacturing facilities of GIA (ECOAQUA Institute, ULPGC, Las Palmas, Spain). The diet (Table 2) was formulated to contain high levels of vegetable oils and thus contained high levels of oleic acid (18:1n-9) (32.3% of total fatty acids, TFA), LA (18:2n-6) (20.3 %TFA), and ALA (18:3n-3) (11.8 %TFA) while containing low concentrations of EPA (2.5 %TFA), DHA (1.7 %TFA), and n-3 LC-PUFA (4.6 %TFA) in order to up-regulate the fatty acid desaturase 2 (*fads2*) gene expression [48].

### 2.2. Blood and Liver Sample Collection and Storage

All broodfish (n = 20) were fasted overnight and anesthetized with 10 ppm clove oil (clove oil:methanol (50:50) in sea water) to collect peripheral blood and liver samples at the end of the feeding trial. After the feeding trial, blood samples were collected from the caudal vein of the GSB broodfish using sterile syringes and the whole blood samples were transferred to EDTA-coated tubes. Whole blood samples were centrifuged at 3000× *g* for 10 min held at (4 °C) to separate blood cells and plasma. The peripheral blood cell samples (PBCs) were stored at −80 °C until RNA extraction. Liver samples were collected from each fish and individually stored in a 1.5-mL Eppendorf tube containing RNA Later (Sigma-Aldrich, USA) and was snap frozen in liquid N_2_ immediately after sampling. The samples were then stored at −80 °C until RNA extraction and analyses.

### 2.3. Molecular Study—RNA Extraction and cDNA Synthesis

Total RNA from PBCs (300–400 µL) and liver samples (60–70 mg) were extracted following the manufacturer’s protocol using the RNeasy Mini Kit (Qiagen) and both blood cells and liver samples were completely homogenized with glass beads using the Tissue Lyzer-II (Qiagen, Hilden, Germany) with TRI Reagent (Sigma-Aldrich, San Luis, USA). Chloroform was added to the homogenized samples and centrifuged at 12,000× *g*, 15 min, 4 °C for clear phase separation. The clear upper aqueous phase containing RNA was mixed with 75% ethanol and transferred into the RNeasy spin column, where total RNA bound to a membrane and RW1 (700 µL) and RPE (500 µL) buffers (Qiagen) were used to wash away contaminants. Total RNA from RNeasy spin column was eluted with 50 μL of RNase-free water. The extracted total RNA quality and quantity were checked using a NanoDrop Spectrophotometer (Thermo Scientific, Wilmington, DE, USA). We used 100 ng RNA for the cDNA synthesis and it was carried out as following the standard protocol and cDNA synthesis was performed using the iScript cDNA Synthesis Kit (Bio-Rad) according to manufacturer’s instructions in the iCycler thermal cycler (Bio-Rad, Hercules, CA, USA). The efficiency of Δ6 desaturase primer was tested with serial dilutions of a cDNA pool (1, 1:10, 1:100, 1:200 and 1:1000). Primers for GSB Δ6 desaturase were redesigned with reference to publications in NCBI (National Center for Biotechnology Information) as follows [30,31,32,33,43]:

Gene—*fads2* (Δ6 desaturase; GenBank: GQ162822.1)
Forward primer sequence (5′ → 3′) GCAGAGCCACAGCAGCAGGGA
Reverse sequence (3′ → 5′) CGGCCTGCGCCTGAGCAGTT

### 2.4. Digital Droplet PCR (ddPCR) Analysis for Absolute Gene Expression

Total RNA extraction from peripheral blood cells and liver were made using a similar protocol described above for the qPCR. Absolute gene expression of PBCs and liver *fads2* analysis were performed using Digital Droplet PCR (ddPCR) (Bio-rad QX200, Hercules, CA, USA) systems, by using cDNA obtained as mentioned in Section 2.3. Sample preparation for ddPCR was carried out as per the manufacturer’s protocol. The master mixes for *fads2* gene were prepared including 10 μL EvaGreen super mix (Bio-rad, Hercules, CA, USA), 0.2 μL F primer (10 pmol/μL), 0.2 μL R primer (10 pmol/μL), 7.6 μL MilQ water, and 2 μL cDNA. Then, droplets were generated using droplet generator Bio-rad QX200 (Hercules, CA, USA) and the droplets were transferred to 96 well microplates for PCR in a thermal cycler (Bio-rad C1000 Touch, Hercules, CA, USA). After PCR amplifications, droplets were measured with a droplet reader (Bio-rad QX200, Hercules, CA, USA) to determine absolute gene expression of *fads2* gene. The samples with less than 12,000 droplets were not used for the gene expression study. The *fads2* gene expression analysis was performed in two replicates for each sample and values were expressed as mRNA copies/µL [31,33].

### 2.5. Liver Fatty Acid Analysis

Liver samples were collected from all the broodfish and stored at −80 °C for analysis of fatty acid composition. Crude lipid extraction was carried out with chloroform:methanol [49]. Hepatic fatty acids methyl esters (FAMES) from total lipids were prepared by transmethylation method with 1% sulfuric acid in methanol [50], purified on NH2 silica (Sep-pak; Waters), and separated and quantified in a gas chromatograph (GC14A; Shimadzu, Kyoto, Japan) equipped with a flame ionization detector and a Carbowax 20 M (30 m × 0.32 mm × 0.27 m) silica capillary column (length: 30 m; internal diameter: 0.32 mm; Supelco, Bellefonte, PA, USA) using helium as a carrier gas. Column initial temperature was set to 170 °C for 10 min and then it was raised to 220 °C at 2.5 °C per min and finally maintained at 215 °C for a further 5 min. FAMES were identified by comparison with previously characterized standards [51]. Specific unclear peaks were identified by GLC-MS (TRACE^TM^ GC Ultra and PolarisQ mass spectrometer; Thermo Fisher Scientific, Spain).

### 2.6. Ethical Statement

The fish broodstock study was conducted as per guidelines of the European Union Directive (2010/63/EU) on the protection of animals for scientific purposes. The study was conducted at Aquaculture Research Group (GIA), ECOAQUA, University of Las Palmas de Gran Canaria (ULPGC), Canary Islands, Spain as per the ethical committee norms. All these samplings and studies were approved by the Bioethical Committee of the ULPGC *vide* REF: 007/2012 CEBA ULPGC.

### 2.7. Statistical Analysis

All results were reported as mean ± standard deviation. All data was checked for normality (Kolmogorov–Smirnoff) and homogeneity (Levene’s tests) and if no normality was observed, an arcsine transformation was performed to attain normality. All of the percentage data were arcsine-transformed before performing statistical analysis. The regression and Pearson’s correlation analysis were performed for studying the relationship between *fads2* expression in PBCs and liver and between sex and broodstock body weight. The independent sample student’s *t*-test was performed to compare male (n = 4) and female (n = 16) broodstock for body weight, hepatosomatic index (HSI %), gonadosomatic index (GSI %), PBCs, liver *fads2* expression, and hepatic fatty acid composition. Pearson’s correlation coefficient was calculated for liver *fads2* expression on hepatic fatty acid of either male or female broodstock. All data was analyzed using the program IBM SPSS version 20 for Windows (IBM SPSS Inc, Armonk, NY, USA).

## 3. Results

### 3.1. Biometric and fads2 Expression Values for Males and Females

**Figure 2:**
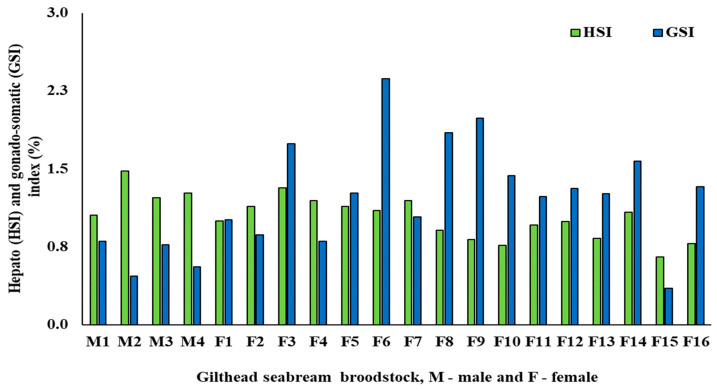
HSI (hepatosomatic index) or GSI (gonadosomatic index) (%) of gilthead seabream male (n = 4) or female (n = 16) broodstock (M1 to M4—Male; F1 to F16—Female).

The GSB male and female broodstock size, HSI %, GSI %, and *fads2* expression in PCBs and liver are presented in Table 3. As expected, mean body length and weight were significantly higher for females than for males (Table 3). The mean HSI values for males (1.26 ± 0.17) was 18% higher (*p* = 0.04) than for females (1.08 ± 0.13) (Table 3), whereas GSI % values for females (1.47 ± 0.36) were about 2.5 times higher (*p* < 0.001) than for males (0.65 ± 0.16) (Table 3). HSI values for each individual fish ranged between 1.06–1.48 and 0.83–1.48% for male and female broodstock, respectively (Figure 2), whereas those for GSI ranged from 0.47–0.80 and from 1.01–2.37% for male and female broodstock, respectively (Figure 2).

The mean PBC *fads2* expression of male and females were 1.68 ± 0.55 and 2.00 ± 0.93 copies/µL, respectively and liver *fads2* expression was 2.60 ± 0.84 and 3.24 ± 1.49 copies/µL for male and female broodstock, respectively (Table 3). There was a very high coefficient of variation in the *fads2* expression levels in both males (32%) and females (46%). The Student *t*-test showed that the *fads2* expression in PBCs or liver was not significantly (*p* > 0.05) different between males and females, although *fads2* values in PBCs and liver were 20 and 25% higher in females than in males (Table 3). No significant correlation was found between body weight and HSI (%) or between body weight and GSI (%) of either male or female broodstock (Table 4).

### 3.2. Comparison of fads2 Expression in PBC or in Liver and Broodstock Body Weight

Expression of *fads2* in PBCs was not correlated to body weight in males (r = −0.56; *p* = 0.45), females (r = 0.15; *p* = 0.58) nor both sexes (r = 0.19; *p* = 0.42) broodstock (Table 5). In males, we found a significant negative correction (r = −0.96; *p* = 0.04) between liver *fads2* expression and body weight, whereas in females liver *fads2* expression did not show any correlation (r = 0.13; *p* = 0.64) to body weight (Table 5). Moreover, we could not find any correlation between broodfish body weight and liver *fads2* expression (r = 0.19; *p* = 0.43) in either sex. Moreover, neither *fads2* expression in PBCs nor in liver showed a significant regression with body weight for males, females or both sexes (*p* > 0.05; data not showed).

### 3.3. Relationship between fads2 Expression in PBCs and in Liver of Male and Female Broodstock

**Figure 3:**
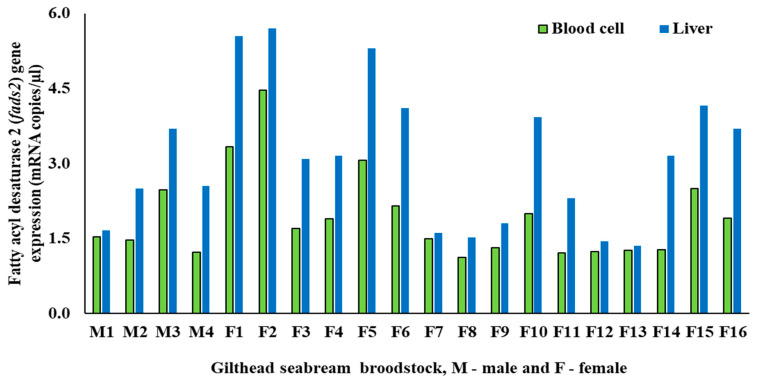
Fatty acyl desaturase 2 (*fads2*) mRNA expression (copies/µl) in PBCs or liver of gilthead seabream male (n = 4) and female (n = 16) broodstock (M1 to M4—male; F1 to F16—female).

**Figure 4:**
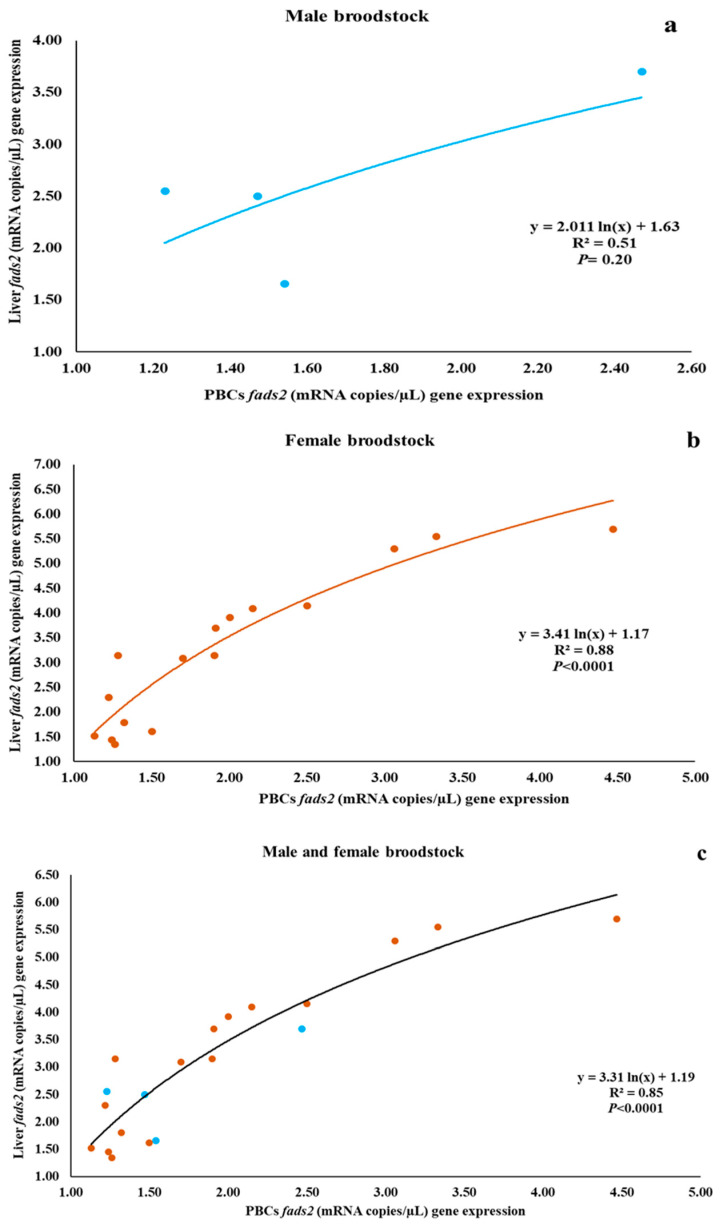
The regression relationship between PBCs and liver *fads2* mRNA expression (copies/µL) in gilthead seabream male (n = 4) (**a**), female (n = 16) (**b**), or both (n = 20) (**c**) broodstock.

Individual data on *fads2* expression in PBCs and liver of male and female broodstock are presented in Figure 3. The *fads2* expression values in PBCs and liver of male broodstock did not significantly (*p* = 0.114) differ (Table 6) and Pearson’s correlation analysis showed a positive (r = 0.76), but not significant (*p* = 0.24), correlation between both parameters (Table 7). On the contrary, *fads2* expression was significantly lower in PBCs compared to the liver for females (*p* = 0.008) and both sexes (*p* = 0.003) (Table 6). Additionally, female broodstock exhibited a highly positive (r = 0.90) and very significant (*p* < 0.001) correlation in the *fads2* expression between PBCs and liver. The same trend was observed for PBCs and liver *fads2* gene expression of pooled results for both male and female broodstock (r = 0.89; *p* < 0.001) (Table 7). The regression relationship analysis results for males and females showed that data for females (R² = 0.88; *p* < 0.001) and for both sexes combined (R² = 0.85; *p* < 0.001) exhibited a significantly higher level of regression between PBCs and liver *fads2* expression than in male broodstock (R² = 0.51; *p* = 0.29) (Figure 4).

### 3.4. Fatty Acid Composition of the Liver

Liver fatty acid profiles were very similar between males and females (Table 8). Thus, mean content in each fatty acid was not significantly different between males and females, except for 16:4n-3 fatty acid, which was 47% higher (*p* = 0.01) in males (Table 8). Consequently, the sum of fatty acids belonging to saturated, monounsaturated, n-9, n-6, or n-3 families were not significantly different between males and females (*p* > 0.05) (Table 8). Similarly, there were no significant differences in the ratios among main essential fatty acids (*p* > 0.05) observed (Table 8). Fatty acid profiles of individual broodfish markedly differed among individuals, including fatty acids of interest in the bioconversion pathway (Supplementary Table S1). For instance, EPA levels in males ranged between 1.5 to 4.5% and in females between 1.5 to 5.0%, whereas DHA levels were higher than those of EPA with individual values ranging between 5.2 to 13.7% in males and between 6.0 to 14.4% in females (Supplementary Table S1). Among the males, the one with the highest liver *fads2* expression (M3) and the lowest weight showed the highest hepatic ARA, DHA, total n-3 LC-PUFA, or EPA+DHA contents (Supplementary Table S1). However, among the females, the one with the highest *fads2* expression (F2) did not show the highest levels of these fatty acids (Supplementary Table S1).

To determine the relationship between liver *fads2* expression and hepatic fatty acid composition, a Pearson’s correlation analysis was conducted (Table 9). In male broodstock, liver *fads2* expression was highly (*p* < 0.05) correlated to 20:3n–6/20:2n–6 (r = 0.98), a *fads2* product/precursor ratio, and slightly (*p* < 0.1) correlated to 20:3n-9 (r = 0.94), 20:3n-6 (r = 0.91), 22:4n-6 (r = 0.91), 22:5n-6 (r = 0.93), and 22:6n-3 (r = 0.92), all of them direct or indirect products from Fads2 activity. In females, none of the hepatic FAs showed significant (*p* > 0.05) correlations with liver *fads2* expression.

## 4. Discussion

Fads2 is a rate-limiting enzyme involved in the first step of LC-PUFA biosynthesis in all vertebrates. In many marine fish, including gilthead seabream (GSB), its activity is low and the expression of the gene responsible for its production (*fads2*) is very low [1,17,30,31]. Fads2 has dual ∆6 or ∆8 activities in many marine fish including GSB [19,32]. An increase in *fads2* expression may result in higher ∆6 Fads production, yielding a higher production of essential fatty acids in fish [31,32]. The implication of the *fads2* gene expression pattern in male and female animals has been studied to elucidate its importance on reproductive performance in higher vertebrates [45,46,47,52,53] and in fish [31,33]. However, there are no previous studies determining the potential relationship between male and female body weight and *fads2* expression in different tissues. As expected from a protandric hermaphrodite species, females showed a significantly higher body weight than males. However, there were no significant relationships between *fads2* expression in PCBs and the body weight of males, females, or both sexes in broodstock observed. These results are in accordance with our previous studies, in which no correlations were found between either parameters [31]. Moreover, there was no significant correlation of body weight with *fads2* expression in the liver of females nor in both sexes in broodstock noticed. However, the highest liver *fads2* expression found in male broodstock with the lowest body weight (M3), which could be related to individual differences in the genome or epigenome, in agreement with our studies [31,33]. These results indicated that there is no relationship between broodstock body weight and the relative expression of *fads2* in PBCs or liver.

Our previous studies on reproductive performance of GSB have found that, during the spawning season, female broodstock shows higher *fads2* expression in PBC compared to male broodstock [31]. Moreover, in GSB females, there is a significant positive correlation between the plasma 17β-estradiol levels and the *fads2* expression in PBC in accordance with studies reported in mammals [45,54,55,56]. In agreement with the present study, *fads2* expression in PBCs and in liver were respectively 20 and 25% higher in females than in males, but were not significantly different. This lack of significance could be due to the fact that the present study was conducted prior to the spawning season, when female gametogenesis was being initiated and GSI still remained low, but was significantly higher than that of males. Therefore, 17β-estradiol levels, directly related to the *fads2* expression in PBC, could be expected to still be relatively low. Indeed, fatty acid profiles of liver in females showed 7–28% higher Fads2-derived fatty acid products from n-9 and n-6 series than in male livers. However, n-3 *fads2*-derived fatty acids tend to be higher in males. These differences among fatty acid families showed that the fatty acid profile of a tissue is not only related to the desaturation activity. For instance, during exogenous vitellogenesis in female liver, there is an increased synthesis of lipoproteins, particularly phosvitin and lipovitelin rich in n-3 LC-PUFA, which transports lipids to the developing oocyte [57]. In agreement with the present study, the GSI was significantly (*p* < 0.001) higher in females than in males, whereas the HSI was 18% lower (*p* = 0.04) than in males, suggesting the mobilization of nutrients from liver to gonads. These results agree with the higher GSI observed in female in comparison to males [58,59,60]. In addition, increased *fads2* expression in male liver led to increased contents of 20:3n–6/20:2n–6, a *fads2* product/precursor ratio, and a slight increase of 20:3n-9, 20:3n-6, 22:4n-6, 22:5n-6, and 22:6n-3, suggesting an increase in lipogenesis pathways. These results agree well with the increased expression of fatty acid synthase genes (*fas*) found in the liver of seabream with increased *fads2* expression [61]. Higher *fads2* expression in GSB females in comparison to males would be justified by their high EFA requirements during vitellogenesis [30,62] to supply vital nutrients to the gamete and embryo [30,63,64,65,66]. Besides, LC-PUFA, including ARA and EPA, are required as precursors for the production of prostaglandins [67] that regulate steroidogenesis, which in turn induce vitellogenin synthesis in liver [68,69,70]. Therefore, the high variability in liver fatty acid profiles of individual brood fish could reflect the dynamic nature of the fatty acid pool in this metabolically active tissue, especially diverting the EFAs towards gametogenesis. Such a specific utilization of EFAs is also in agreement with the suggestions on age- or size-related increases in requirements for essential fatty acids shown in rodents [71,72,73,74] and fish [75].

The high correlation found in broodstock males between liver *fads2* expression and Fads2 products or product/precursor ratio was in agreement with the positive correlation between liver *fads2* mRNA expression and 18:3n-6/18:2n-6 ratio found also in rats [76]. Besides, the positive correlation between 20:3n-6 (dihomo-γ-linolenic acid, DGLA) and ARA with PBCs and/or liver, *fads2* expression points out the importance of both fatty acids as precursors of series-1 and series-2 prostaglandins, particularly PGE1 and PGE2, which act as precursors and are involved in cell signaling, hormone production, and as an anti-inflammatory molecule [67,77,78,79]. In agreement with our results, peripheral blood FADS2 expression has been shown to have a positive correlation to DGLA fatty acids in humans [80]. Prostaglandins derived from both fatty acids are found to have a positive effect on testosterone production and sperm quality in fish [81,82,83].

In GSB broodstock, *fads2* expression in PBCs has been associated with reproductive performance, suggesting that PBCs *fads2* expression could be a useful biomarker to be considered for broodstock selection [31,32,33]. For instance, GSB expressing higher levels of PBCs *fads2* produces higher amounts of eggs and larvae, as well as juveniles with an improved utilization of low fish meal and fish oil diets [30,31,32,33,43]. Additionally, we have observed (unpublished) that male broodstock showing higher PBCs *fads2* expression also have a better sperm quality with increased sperm motility and duration. Peripheral blood mononuclear cell (PBMCs) gene expression has been proposed as a potential biomarker to correlate the expression of the same genes in various tissues that are non-accessible for biopsies to predict health status, physiological condition, stress, disease diagnosis, or nutrient metabolism in human and land animals [35,37,84,85,86,87]. In GSB, PBCs have also been studied as a non-invasive tool to assess mitochondrial energy metabolism [39]. However, until now, the possible relationship between liver *fads2* and PBC *fads2* expression has not been addressed. Our study demonstrated that PBC *fads2* expression is directly related to liver *fads2* expression, since the expression in both types of cells is approximately equal in the males and no correlation was found. On the other hand, a clear correlation between PBCs and liver *fads2* expression was found in female GSB broodstock and was highly correlated in females. Therefore, these results confirm that PBC *fads2* expression is a relevant non-invasive biomarker to undertake marker-based selection for improved production of EFAs and reproductive performance in fish. Therefore, PBCs *fads2* expression may be a good non-invasive indicator to study the role of *fads2* in growth, body composition, health, or reproduction, as well as in studies on fatty acid biosynthesis and metabolism in fish and other animals.

## 5. Conclusions

This study aimed to find the relationship between *fads2* expression patterns in peripheral blood cells and liver of broodstock gilthead seabream, *Sparus aurata*, to see the possible use of the *fads2* gene as a potential biomarker for the selection of broodstock to undertake breeding programs aiming at improved reproduction, health and nutritional status. We found a highly positive correlation between *fads2* expression levels in the PBCs and liver of GSB broodstock. PBC *fads2* can be utilized as a valid biomarker for fatty acid metabolism in fish and is applicable to broodstock selection programs. PBC *fads2* expression levels have a good potential as a non-invasive method to select animals having increased fatty acid bioconversion capability and a better ability to deal with fish meal and fish oil free diets.

## Figures and Tables

**Table 1:** Ingredients (%) and proximate composition of the low fish meal and low fish oil diet fed to gilthead seabream broodstock.

Ingredients (%)	Experimental Diet
Fish meal (South American)	5.00
Blood meal (spray-dried)	7.00
Soya protein concentrate	20.00
Corn gluten meal	22.00
Wheat gluten	5.50
Rapeseed meal	11.30
Wheat	6.89
Fish oil (South American)	3.00
Linseed oil	2.60
Palm oil	5.20
Rapeseed oil	5.20
Supplemented ingredients ^1^	5.49
Vitamin and mineral premix ^2^	0.75
Antioxidant-Ethoxyquin	0.05
Yttrium oxide	0.03
**Proximate Composition**	
Crude protein (% dry matter, DM)	45.1
Crude lipid (% DM)	21.7
Ash (% DM)	5.4
Moisture (%)	9.0

^1^ Supplemented ingredients contain—lysine, methionine, monocalcium phosphate, choline, inositol, phospholipids. ^2^ Vitamin and mineral premix—vitamins (mg/kg): A 3.8, D 0.05, E 102.4, K3 9.8, B1 2.7, B2 8.3, B6 4.8, B12 0.25, B3 24.8, B5 17.2, folic acid 2.8, H 0.14, C 80; minerals (mg/kg): cobalt 0.94, iodine 0.7, selenium 0.2, iron 32.6. manganese 12, copper 3.2, zinc 67; other (g/kg): taurine 2.45, methionine 0.5, histidine 1.36, cholesterol 1.13.

**Table 2:** Fatty acid profiles of low fish meal and fish oil diet for gilthead seabream broodstock (% total fatty acids).

Fatty Acid (%TFA)	Experimental Diet
14:0	6.6
14:1n-5	0.1
15:0	0.1
16:0	12.3
16:1n-7	2.1
16:1n-5	0.1
16:2n-4	0.2
17:0	0.3
16:3n-4	0.1
16:4n-3	0.4
18:0	3.2
18:1n-9	32.3
18:1n-7	2.3
18:2n-6 (LA)	20.3
18:2n-4	0.1
18:3n-6	0.1
18:3n-3 (ALA)	11.8
18:4n-3	0.4
20:0	0.4
20:1n-9	1.0
20:1n-7	0.1
20:2n-6	0.1
20:4n-6 (ARA)	0.2
20:4n-3	0.1
20:5n-3 (EPA)	2.5
22:1n-11	0.1
22:1n-9	0.3
22:5n-6	0.1
22:5n-3 (DPA)	0.3
22:6n-3 (DHA)	1.7
Total saturates	22.9
Total monoenes	38.4
Total n-3	17.2
Total n-6	20.8
Total n-9	32.6
Total n-3 LC-PUFA	4.6

**Table 3:** Gilthead seabream male and female broodstock body length (cm), weight (kg), HSI %, GSI %, and PBCs and liver *fads2* expression (mRNA copies/µL) after feeding the low fish meal and fish oil diet for one month. Superscript “a” or “b” indicates significantly higher or lower mean value, respectively.

Broodstock	Males (n = 4)	Female (n = 16)	*t*-Test (*p*-Value)
Body length (cm)	47 ± 3 ^b^	56 ± 4 ^a^	<0.001
Body weight (kg)	1.27 ± 0.19 ^b^	2.40 ± 0.63 ^a^	0.003
HSI (%)	1.26 ± 0.17 ^a^	1.08 ± 0.13 ^b^	0.04
GSI (%)	0.65 ± 0.16 ^b^	1.47 ± 0.36 ^a^	<0.001
PBCs *fads2* (mRNA copies/µL)	1.68 ± 0.55	2.00 ± 0.93	0.52
Liver *fads2* (mRNA copies/µL)	2.60 ± 0.84	3.24 ± 1.49	0.43

Different superscripts in each row indicate significant differences among male or female broodfish (*p* < 0.05, Independent Sample student’s *t*-test). HSI (%) (hepatosomatic index) = (Liver weight, g/weight of fish, g) × 100; GSI (%) (gonadosomatic index) = (Gonad weight, g/weight of fish, g) × 100.

**Table 4:** Pearson’s correlation of body weight (kg) and HSI or GSI (%) of male and female gilthead seabream broodstock.

Broodstock	Male Broodstock		Female Broodstock	
	HSI (%)	GSI (%)	HSI (%)	GSI (%)
Body weight (kg)	−0.35 (0.65)	0.14 (0.86)	0.14 (0.61)	0.25 (0.36)

**Table 5:** Pearson’s correlation coefficient (PC) of body weight (kg) and PBCs or liver *fads2* expression (mRNA copies/µL) of male, female, and both male and female gilthead seabream broodstock.

Broodstock	Male Broodstock		Female Broodstock		Male and Female Broodstock	
	PBCs *fads2*	Liver *fads2*	PBCs *fads2*	Liver *fads2*	PBCs *fads2*	Liver *fads2*
Body weight (kg)	−0.56 (0.45)	−0.96 (0.04)	0.15 (0.58)	0.13 (0.64)	0.19 (0.42)	0.19 (0.43)

**Table 6:** Independent sample student’s *t*-test of PBCs and liver *fads2* expression (mRNA copies/µL) in male, female, and both male and female gilthead seabream broodstock.

Broodstock	PBCs *fads2*	Liver *fads2*	*t*-Test (*p*-Value)
Male	1.68 ± 0.55	2.60 ± 0.84	0.114
Female	2.00 ± 0.93 ^b^	3.24 ± 1.49 ^a^	0.008
Male and female	1.93 ± 0.87 ^b^	3.11 ± 1.39 ^a^	0.003

Different superscripts in each row indicate significant differences (*p* < 0.05, Independent sample student’s *t*-test).

**Table 7:** Pearson’s correlation coefficient (PC) of PBCs and liver *fads2* expression (mRNA copies/µL) in male, female, and both male and female gilthead seabream broodstock.

	Male Broodstock	Female Broodstock	Male and Female Broodstock
	**Liver *fads2***		
**PBCs *fads2***	0.76 (0.24)	0.90 (<0.001)	0.89 (<0.001)

**Table 8:** Mean hepatic fatty acid composition (% total fatty acids) of male or female gilthead seabream broodstock after feeding the low fish meal and fish oil diet for one month.

Fatty Acid (%TFA)	Male		Female		*t*-Test (*p*-Value)
	Mean	SD	Mean	SD	
14:0	1.42	0.57	1.30	0.38	0.59
14:1n-5	0.04	0.02	0.03	0.01	0.33
14:1n-7	0.02	0.01	0.03	0.01	0.70
15:0	0.17	0.03	0.14	0.04	0.19
15:1n-5	0.02	0.01	0.02	0.01	0.82
16:0	11.56	0.29	11.41	1.42	0.72
16:1n-7	2.48	1.12	2.18	0.46	0.64
16:1n-5	0.06	0.02	0.05	0.01	0.43
16:2n-4	0.14	0.11	0.10	0.04	0.48
17:0	0.14	0.08	0.11	0.04	0.35
16:3n-4	0.16	0.03	0.14	0.02	0.30
16:3n-3	0.06	0.02	0.05	0.01	0.40
16:3n-1	0.04	0.04	0.03	0.01	0.76
16:4n-3	0.17 ^a^	0.06	0.09 ^b^	0.04	0.01
18:1n-9	3.86	0.95	3.80	0.45	0.66
18:1n-7	28.81	3.98	29.80	1.61	0.67
18.1n-5	2.69	0.35	2.61	0.15	0.76
18:2n-9	0.11	0.03	0.10	0.03	0.30
18:2n-6 (LA)	0.09	0.05	0.11	0.04	0.71
18:2n-4	14.51	2.52	14.86	1.41	0.77
18:3n-6	0.09	0.04	0.09	0.02	0.61
18:3n-4	0.24	0.07	0.25	0.06	0.56
18:3n-3 (ALA)	0.13	0.03	0.12	0.03	0.43
18.3n-1	9.01	3.24	9.92	1.68	0.45
18:4n-3	0.01	0.01	0.00	0.01	0.72
18:4n-1	0.55	0.29	0.49	0.13	0.82
20:0	0.09	0.04	0.08	0.03	0.45
20:1n-9	0.29	0.02	0.26	0.06	0.71
20:1n-7	0.36	0.18	0.33	0.11	0.66
20.1n-5	2.34	0.72	2.22	0.42	0.86
20:2n-9	0.15	0.05	0.15	0.06	0.24
20:2n-6	0.18	0.05	0.23	0.08	0.34
20:3n-9	0.86	0.18	0.94	0.15	0.88
20:3n-6	0.03	0.01	0.03	0.01	0.60
20:4n-6 (ARA)	0.26	0.16	0.29	0.10	0.74
20:3n-3	0.77	0.64	0.66	0.18	0.17
20:4n-3	0.78	0.28	0.95	0.19	0.76
20:5n-3 (EPA)	0.66	0.17	0.68	0.13	0.75
22:1n-11	2.80	1.26	2.61	1.00	0.50
22:1n-9	1.48	0.92	1.22	0.62	0.97
22:4n-6	0.65	0.18	0.65	0.18	0.35
22:5n-6	0.23	0.13	0.18	0.06	0.56
22:5n-3 (DPA)	0.19	0.08	0.18	0.05	0.66
22:6n-3 (DHA)	2.04	0.78	1.88	0.57	0.61
Total Saturates	9.32	3.73	8.57	2.30	0.66
Total Monoenes	17.43	0.73	17.02	1.77	0.90
Total n-3	39.20	5.71	39.40	1.90	0.94
Total n-6	25.37	3.44	25.24	2.76	0.86
Total n-9	17.05	3.08	17.36	1.39	0.64
Total n-3 LC-PUFA	30.11	4.01	31.15	1.38	0.69
EPA+DHA	15.59	5.04	14.69	3.68	0.62
ARA/EPA	12.12	4.44	11.18	3.11	0.82
EPA/ARA	0.31	0.27	0.28	0.12	0.64
DHA/ARA	5.01	3.05	4.19	1.69	0.66
DHA/EPA	14.72	5.44	13.36	2.97	0.83
DHA/DPA	3.57	1.48	3.46	0.80	0.88
n-3/n-6	4.82	2.20	4.64	0.84	0.74
n-6/n-3	1.52	0.32	1.47	0.26	0.78
18:2n-9/18:1n-9	0.68	0.13	0.70	0.10	0.79
18:3n-6/18:2n-6	0.66	0.54	0.75	0.34	0.86
20:3n-6/20:2n-6	0.02	0.01	0.02	0.01	0.71
18:4n-3/18:3n3	0.29	0.12	0.31	0.09	0.52
20:4n-3/20:3n-3	0.08	0.06	0.05	0.03	0.48

Different superscripts in each row indicate significant differences among male or female broodfish hepatic fatty acid composition (*p* < 0.05, Independent sample student’s *t*-test).

**Table 9:** Pearson’s correlation of liver *fads2* expression (mRNA copies/µL) and hepatic fatty acid (%TFA) composition of male and female gilthead seabream broodstock.

Fatty Acids (%TFA)	Male Broodstock		Female Broodstock	
	Pearson’s Correlation	*p*-Value	Pearson’ Correlation	*p*-Value
14:0	−0.57	0.43	−0.07	0.79
14:1n-5	−0.58	0.42	−0.22	0.42
14:1n-7	−0.89	0.11	−0.12	0.66
15:0	−0.57	0.43	−0.20	0.45
15:1n-5	−0.43	0.57	−0.33	0.21
16:0	0.95	0.04	0.25	0.35
16:1n-7	−0.40	0.60	−0.13	0.62
16:1n-5	−0.11	0.89	−0.04	0.88
16:2n-4	−0.37	0.63	−0.17	0.52
17:0	−0.15	0.85	−0.31	0.24
16:3n-4	−0.70	0.30	0.04	0.87
16:3n-3	−0.11	0.89	−0.33	0.22
16:3n-1	0.85	0.15	−0.48	0.06
16:4n-3	0.32	0.68	−0.19	0.48
18:0	0.83	0.17	0.45	0.08
18:1n-9	−0.75	0.25	0.28	0.29
18:1n-7	−0.42	0.58	0.03	0.90
18.1n-5	−0.10	0.90	−0.19	0.48
18:2n-9	0.86	0.14	0.05	0.85
18:2n-6 (LA)	−0.10	0.90	0.11	0.69
18:2n-4	−0.11	0.89	−0.39	0.14
18:3n-6	0.93	0.07	−0.04	0.89
18:3n-4	−0.11	0.89	−0.14	0.61
18:3n-3 (ALA)	−0.37	0.63	0.10	0.72
18.3n-1	−0.11	0.89	0.20	0.46
18:4n-3	−0.16	0.84	−0.22	0.41
18:4n-1	−0.39	0.61	−0.34	0.20
20:0	0.14	0.86	−0.35	0.18
20:1n-9	−0.40	0.60	−0.24	0.38
20:1n-7	−0.44	0.56	−0.29	0.29
20.1n-5	−0.27	0.73	−0.17	0.52
20:2n-9	0.86	0.14	0.28	0.29
20:2n-6	0.49	0.51	0.19	0.49
20:3n-9	0.94	0.07	−0.14	0.60
20:3n-6	0.91	0.09	0.23	0.40
20:4n-6 (ARA)	0.89	0.11	−0.03	0.91
20:3n-3	0.31	0.69	0.30	0.26
20:4n-3	0.62	0.38	−0.03	0.92
20:5n-3 (EPA)	0.20	0.80	−0.29	0.27
22:1n-11	−0.25	0.75	−0.29	0.27
22:1n-9	−0.33	0.67	−0.12	0.65
22:4n-6	0.91	0.09	−0.06	0.83
22:5n-6	0.93	0.07	−0.11	0.69
22:5n-3 (DPA)	0.12	0.88	−0.21	0.45
22:6n-3 (DHA)	0.92	0.08	−0.21	0.44
Total Saturates	0.97	0.03	0.28	0.30
Total Monoenes	−0.76	0.24	0.01	0.96
Total n-3	0.80	0.20	−0.26	0.34
Total n-6	0.26	0.74	0.14	0.62
Total n-9	−0.76	0.25	0.31	0.24
Total n-3 LC-PUFA	0.79	0.21	−0.23	0.40
EPA + DHA	0.83	0.17	−0.25	0.36
ARA/EPA	0.72	0.28	0.14	0.61
EPA/ARA	−0.40	0.60	−0.25	0.35
DHA/ARA	−0.45	0.55	−0.21	0.44
DHA/EPA	0.69	0.31	0.20	0.46
DHA/DPA	0.81	0.19	−0.02	0.94
n-3/n-6	0.24	0.76	−0.27	0.31
n-6/n-3	−0.38	0.62	0.21	0.43
18:2n-9/18:1n-9	0.87	0.13	−0.04	0.90
20:2n-9/20:1n-9	0.89	0.11	0.30	0.27
18:3n-6/18:2n-6	0.75	0.25	−0.32	0.23
20:3n-6/20:2n-6	0.98	0.02	0.15	0.58
18:4n-3/18:3n3	−0.04	0.96	−0.15	0.58
20:4n-3/20:3n-3	0.05	0.95	−0.26	0.32

## Supplementary Materials

The following are available online at https://www.mdpi.com/2075-1729/10/7/117/s1, Table S1: Hepatic fatty acid composition (% total fatty acids) of male and female gilthead sea bream broodstock fed a low FM and low FO diet for one month (M1 to M4—Male; F1 to F16—Female).

## Abbreviations

ALA	linolenic acid (18:3n-3)
ARA	arachidonic acid (20:4n-6)
ddPCR	droplet digital polymerase chain reaction
DHA	docosahexaenoic acid (22:6n-3)
EPA	eicosapentaenoic acid (20:5n-3)
*fads2*	fatty acyl desaturase 2 (gene in fish)
FADS	fatty acyl desaturase (enzyme in mammals)
Fads	fatty acyl desaturase (enzyme in fish)
FM	fish meal
FO	fish oil
GSB	gilthead seabream
HD	high *fads2* expression
LA	linoleic acid (18:2n-6)
LC-PUFA	long chain polyunsaturated fatty acid
LD	low *fads2* expression
PBCs	peripheral blood cells
TFA	total fatty acids
