# Supplementary material for: The Relationship between the Expression of Fatty Acyl Desaturase 2 (fads2) Gene in Peripheral Blood Cells (PBCs) and Liver in Gilthead Seabream, Sparus aurata Broodstock Fed a Low n-3 LC-PUFA Diet
Source: Life (Basel). 2020 Jul 19;10(7):117. doi: 10.3390/life10070117 (PMC7400341; doi:10.3390/life10070117)
Supplement: Supplementary file 1 [file life-10-00117-s001.pdf]

Supplementary

Table S1: Hepatic fatty acid composition (% total fatty acids) of male and female gilthead sea bream broodstock fed a low FM and low FO diet for one month (M1 to M4—Male; F1 to F16—Female).

**Fatty Acid (%TFA)**	**M1**	**M2**	**M3**	**M4**	**F1**	**F2**	**F3**	**F4**	**F5**	**F6**	**F7**	**F8**	**F9**	**F10**	**F11**	**F12**	**F13**	**F14**	**F15**	**F16**
14:0	1.38	2.10	0.71	1.50	0.97	1.75	2.02	1.45	1.01	1.86	1.43	1.37	1.08	0.68	1.25	1.68	0.93	1.18	1.05	1.03
14:1n-5	0.04	0.06	0.02	0.04	0.02	0.04	0.05	0.03	0.03	0.05	0.04	0.04	0.02	0.02	0.02	0.04	0.05	0.02	0.03	0.03
14:1n-7	0.03	0.03	0.01	0.02	0.02	0.03	0.04	0.03	0.02	0.03	0.02	0.02	0.02	0.01	0.01	0.02	0.05	0.04	0.01	0.03
15:0	0.17	0.21	0.13	0.16	0.10	0.17	0.18	0.17	0.10	0.19	0.16	0.17	0.13	0.09	0.10	0.19	0.11	0.12	0.15	0.13
15:1n-5	0.03	0.02	0.02	0.01	0.02	0.02	0.02	0.02	0.01	0.02	0.02	0.02	0.02	0.01	0.01	0.02	0.05	0.04	0.02	0.02
16:0 *iso*	0.00	0.04	0.02	0.03	0.01	0.03	0.03	0.00	0.02	0.03	0.02	0.02	0.02	0.02	0.01	0.02	0.04	0.02	0.02	0.02
16:0	11.31	11.54	11.96	11.41	12.59	12.40	12.70	13.72	11.85	11.53	11.96	10.55	12.51	10.12	9.76	12.75	8.25	10.47	10.27	11.20
16:1n-7	2.00	3.77	1.19	2.94	1.93	2.56	2.72	1.80	2.01	3.24	2.38	2.39	1.75	1.44	2.46	2.47	2.35	1.98	1.74	1.73
16:1n-5	0.04	0.07	0.04	0.07	0.04	0.06	0.06	0.03	0.05	0.06	0.05	0.05	0.05	0.04	0.03	0.05	0.06	0.04	0.04	0.04
16:2n-4	0.10	0.28	0.03	0.16	0.07	0.12	0.15	0.06	0.09	0.17	0.12	0.14	0.06	0.05	0.04	0.12	0.15	0.09	0.09	0.08
17:0	0.08	0.25	0.07	0.14	0.07	0.11	0.14	0.07	0.08	0.14	0.10	0.12	0.08	0.06	0.11	0.12	0.16	0.21	0.09	0.08
16:3n-4	0.16	0.18	0.12	0.17	0.13	0.18	0.17	0.12	0.13	0.18	0.16	0.15	0.13	0.12	0.11	0.16	0.15	0.12	0.15	0.14
16:3n-3	0.04	0.08	0.04	0.06	0.04	0.05	0.06	0.04	0.04	0.06	0.05	0.06	0.04	0.04	0.04	0.04	0.08	0.05	0.04	0.05
16:3n-1	0.02	0.03	0.10	0.01	0.02	0.02	0.02	0.03	0.03	0.02	0.03	0.03	0.05	0.04	0.03	0.03	0.06	0.04	0.04	0.04
16:4n-3	0.12	0.26	0.18	0.13	0.07	0.12	0.13	0.05	0.08	0.15	0.10	0.13	0.06	0.05	0.03	0.12	0.17	0.06	0.08	0.08
18:0	3.44	3.08	5.23	3.67	4.42	3.70	4.08	3.87	4.14	3.10	3.48	3.09	4.42	4.28	3.85	3.46	3.09	3.69	3.88	4.17
18:1n-9	31.85	26.36	24.50	32.52	31.34	31.65	30.31	29.40	29.88	29.91	31.28	30.96	28.90	28.06	30.42	31.06	25.57	27.84	30.93	29.36

18:1n-7	2.55	3.08	2.28	2.86	2.62	2.76	2.72	2.36	2.60	2.93	2.66	2.75	2.46	2.45	2.48	2.65	2.71	2.66	2.50	2.44
18:1n-5	0.08	0.15	0.08	0.11	0.08	0.12	0.10	0.08	0.09	0.13	0.10	0.11	0.08	0.08	0.06	0.10	0.18	0.10	0.08	0.11
18:2n-9	0.03	0.07	0.13	0.12	0.10	0.08	0.11	0.05	0.15	0.12	0.06	0.07	0.17	0.12	0.18	0.09	0.12	0.11	0.14	0.10
18:2n-6 (LA)	17.15	11.43	15.85	13.62	14.98	15.18	14.17	17.12	14.21	13.33	15.44	15.53	15.21	14.85	14.83	15.73	11.12	13.76	16.84	15.47
18:2n-4	0.06	0.14	0.06	0.11	0.06	0.09	0.10	0.06	0.08	0.11	0.10	0.10	0.07	0.07	0.06	0.10	0.15	0.09	0.08	0.09
18:3n-6 (GLA)	0.14	0.22	0.31	0.27	0.22	0.18	0.26	0.16	0.29	0.27	0.17	0.20	0.39	0.29	0.23	0.24	0.29	0.26	0.33	0.27
18:3n-4	0.10	0.17	0.10	0.14	0.10	0.12	0.11	0.10	0.12	0.16	0.12	0.14	0.07	0.09	0.08	0.12	0.19	0.13	0.12	0.11
18:3n-3 (ALA)	13.25	5.66	9.54	7.58	10.51	9.90	9.34	13.12	9.22	6.74	10.52	10.31	9.85	10.31	8.86	11.01	6.47	9.48	12.04	11.06
18:4n-3	0.33	0.91	0.29	0.66	0.35	0.49	0.57	0.26	0.51	0.68	0.46	0.55	0.51	0.45	0.38	0.48	0.78	0.42	0.57	0.35
18:4n-1	0.07	0.14	0.04	0.10	0.06	0.09	0.09	0.06	0.06	0.12	0.08	0.10	0.05	0.06	0.05	0.11	0.17	0.08	0.07	0.08
20:0	0.28	0.32	0.29	0.27	0.29	0.21	0.26	0.28	0.24	0.25	0.24	0.27	0.25	0.24	0.22	0.25	0.48	0.27	0.19	0.29
20:1n-9	0.28	0.55	0.15	0.44	0.28	0.33	0.36	0.21	0.31	0.48	0.32	0.39	0.22	0.25	0.28	0.30	0.61	0.45	0.24	0.24
20:1n-7	2.11	3.22	1.50	2.53	2.29	2.08	2.18	1.64	2.13	2.74	2.45	2.51	1.74	2.11	2.42	2.05	3.25	2.41	1.56	1.93
20:1n-5	0.12	0.21	0.10	0.16	0.15	0.14	0.15	0.10	0.13	0.16	0.13	0.14	0.11	0.14	0.16	0.13	0.27	0.32	0.10	0.12
20:2n-9	0.10	0.18	0.22	0.21	0.31	0.16	0.21	0.11	0.37	0.26	0.14	0.15	0.30	0.34	0.25	0.18	0.28	0.22	0.14	0.23
20:2n-6	0.91	0.71	1.08	0.72	1.07	0.84	0.77	0.89	1.01	0.89	1.00	0.88	1.10	1.33	1.02	0.80	0.81	0.77	0.86	1.00
20:3n-9	0.01	0.03	0.04	0.02	0.02	0.03	0.02	0.01	0.03	0.02	0.02	0.02	0.02	0.02	0.02	0.02	0.07	0.05	0.02	0.03
20:3n-6	0.16	0.18	0.50	0.19	0.36	0.18	0.25	0.19	0.48	0.25	0.19	0.15	0.42	0.48	0.34	0.22	0.35	0.28	0.23	0.31
20:4n-6 (ARA)	0.41	0.56	1.72	0.40	0.52	0.44	0.48	0.70	0.75	0.56	0.50	0.47	0.98	1.00	0.76	0.50	0.68	0.65	0.70	0.81
20:3n-3	0.94	0.48	1.08	0.62	1.09	0.84	0.80	0.97	1.01	0.74	0.95	0.85	1.17	1.44	0.83	0.79	0.66	0.89	1.03	1.09

20:4n-3	0.41	0.82	0.71	0.68	0.68	0.57	0.63	0.41	0.82	0.78	0.57	0.65	0.72	0.81	0.81	0.55	0.93	0.73	0.58	0.63
20:5n-3 (EPA)	1.49	4.51	2.41	2.78	1.77	2.23	2.33	1.44	2.46	2.98	2.24	2.57	2.03	2.43	3.31	2.06	5.00	4.82	2.06	2.01
22:1n-11	0.94	2.68	0.61	1.70	0.99	1.10	1.33	0.68	1.15	1.74	1.24	1.52	0.68	0.90	0.85	0.96	3.20	1.58	0.75	0.91
22:1n-9	0.56	0.86	0.46	0.73	0.68	0.55	0.65	0.45	0.71	0.70	0.61	0.63	0.53	0.70	0.53	0.49	1.17	0.93	0.44	0.60
22:4n-6	0.13	0.21	0.42	0.14	0.20	0.14	0.15	0.13	0.18	0.15	0.13	0.15	0.20	0.19	0.16	0.13	0.32	0.24	0.16	0.31
22:5n-6	0.12	0.21	0.29	0.15	0.15	0.14	0.15	0.14	0.21	0.17	0.14	0.13	0.19	0.23	0.18	0.13	0.31	0.19	0.16	0.18
22:5n-3 (DPA)	1.27	3.08	1.69	2.11	1.52	1.75	1.74	1.07	1.90	2.57	1.71	1.96	1.63	2.14	1.86	1.45	3.65	1.88	1.64	1.68
22:6n-3 (DHA)	5.19	10.86	13.69	7.54	6.68	6.25	7.10	6.31	9.16	9.20	6.31	7.40	9.52	11.34	10.51	6.03	14.44	9.82	7.73	9.32
Total Saturates	16.66	17.50	18.39	17.15	18.44	18.34	19.38	19.56	17.42	17.07	17.37	15.57	18.47	15.47	15.29	18.45	13.02	15.94	15.63	16.90
Total Monoenes	40.63	41.06	30.96	44.13	40.46	41.44	40.69	36.83	39.12	42.19	41.30	41.53	36.58	36.21	39.73	40.34	39.52	38.41	38.44	37.56
Total n-3	23.04	26.66	29.63	22.16	22.71	22.20	22.70	23.67	25.20	23.90	22.91	24.48	25.53	29.01	26.63	22.53	32.18	28.15	25.77	26.27
Total n-6	19.02	13.52	20.17	15.49	17.50	17.10	16.23	19.33	17.13	15.62	17.57	17.51	18.49	18.37	17.52	17.75	13.88	16.15	19.28	18.35
Total n-9	32.83	28.05	25.50	34.04	32.73	32.80	31.66	30.23	31.45	31.49	32.43	32.22	30.14	29.49	31.68	32.14	27.82	29.60	31.91	30.56
Total n-3 LC-PUFA	9.30	19.75	19.58	13.73	11.74	11.64	12.60	10.20	15.35	16.27	11.78	13.43	15.07	18.16	17.32	10.88	24.68	18.14	13.04	14.73
EPA+DHA	6.68	15.37	16.10	10.32	8.45	8.48	9.43	7.75	11.62	12.18	8.55	9.97	11.55	13.77	13.82	8.09	19.44	14.64	9.79	11.33
ARA/EPA	0.28	0.12	0.71	0.14	0.29	0.20	0.21	0.49	0.30	0.19	0.22	0.18	0.48	0.41	0.23	0.24	0.14	0.13	0.34	0.40
EPA/ARA	3.63	8.05	1.40	6.95	3.40	5.07	4.85	2.06	3.28	5.32	4.48	5.47	2.07	2.43	4.36	4.12	7.35	7.42	2.94	2.48
DHA/ARA	12.66	19.39	7.96	18.85	12.85	14.20	14.79	9.01	12.21	16.43	12.62	15.74	9.71	11.34	13.83	12.06	21.24	15.11	11.04	11.51
DHA/EPA	3.48	2.41	5.68	2.71	3.77	2.80	3.05	4.38	3.72	3.09	2.82	2.88	4.69	4.67	3.18	2.93	2.89	2.04	3.75	4.64
DHA/DPA	4.09	3.53	8.10	3.57	4.39	3.57	4.08	5.90	4.82	3.58	3.69	3.78	5.84	5.30	5.65	4.16	3.96	5.22	4.71	5.55

n-3/n-6	1.21	1.97	1.47	1.43	1.30	1.30	1.40	1.22	1.47	1.53	1.30	1.40	1.38	1.58	1.52	1.27	2.32	1.74	1.34	1.43
n-6/n-3	0.83	0.51	0.68	0.70	0.77	0.77	0.71	0.82	0.68	0.65	0.77	0.72	0.72	0.63	0.66	0.79	0.43	0.57	0.75	0.70
20:2n-9/20:1n-9	0.36	0.33	1.47	0.48	1.11	0.48	0.58	0.52	1.19	0.54	0.44	0.38	1.36	1.36	0.89	0.60	0.46	0.49	0.58	0.96
18:3n-6/18:2n-6	0.01	0.02	0.02	0.02	0.01	0.01	0.02	0.01	0.02	0.02	0.01	0.01	0.03	0.02	0.02	0.02	0.03	0.02	0.02	0.02
20:3n-6/20:2n-6	0.18	0.25	0.46	0.26	0.34	0.21	0.32	0.21	0.48	0.28	0.19	0.17	0.38	0.36	0.33	0.28	0.43	0.36	0.27	0.31
18:4n-3/18:3n3	0.02	0.16	0.03	0.09	0.03	0.05	0.06	0.02	0.06	0.10	0.04	0.05	0.05	0.04	0.04	0.04	0.12	0.04	0.05	0.03
20:4n-3/20:3n-3	0.44	1.71	0.66	1.10	0.62	0.68	0.79	0.42	0.81	1.05	0.60	0.76	0.62	0.56	0.98	0.70	1.41	0.82	0.56	0.58
